# Supplementary material for: “A very first clue on the subject”: A focus group study on users’ perspectives on German plain language summaries of psychological meta-analyses
Source: PLoS One. 2026 Mar 10;21(3):e0343625. doi: 10.1371/journal.pone.0343625 (PMC12974800; doi:10.1371/journal.pone.0343625)
Supplement: S2 Table — (PDF) [file pone.0343625.s002.pdf]

S2_Table

GRIPP (Guidance for Reporting Involvement of Patients and the Public) 2 Short Form Reporting checklist

Section and topic	Item	Reported on page No
1: Aim	Report the aim of patient and public involvement (PPI) in the study	Background section: 1.1 Problem Formulation, 1.2 Research Question
2: Methods	Provide a clear description of the methods used for PPI in the study	Method section: 2.1 Study design, 2.2 Setting and participants, 2.3 Procedure
3: Study results	Outcomes—Report the results of PPI in the study, including both positive and negative outcomes	Result section: 3.2 Users' aims, 3.3 Users' requirements for characteristics
4: Discussion and conclusions	Outcomes—Comment on the extent to which PPI influenced the study overall. Describe positive and negative effects	Discussion section: 4.2 Main findings in context, 4.3 Strengths and Limitations

5: Reflections/critical perspective	Comment critically on the study, reflecting on the things that went well and those that did not, so others can learn from this experience	Discussion section: 4.3 Strengths and Limitations

From: GRIPP2 reporting checklists: tools to improve reporting of patient and public involvement in research
