# Supplementary material for: “A very first clue on the subject”: A focus group study on users’ perspectives on German plain language summaries of psychological meta-analyses
Source: PLoS One. 2026 Mar 10;21(3):e0343625. doi: 10.1371/journal.pone.0343625 (PMC12974800; doi:10.1371/journal.pone.0343625)
Supplement: S1 File — (PDF) [file pone.0343625.s003.pdf]

Wirkt sich das Spielen von Action-Videospielen auf die Wahrnehmung, die Aufmerksamkeit und das Denken aus?

Eine Übersichtsarbeit

Diese Zusammenfassung bezieht sich auf die Übersichtsarbeit mit dem Titel “Meta-analysis of action video game impact on perceptual, attentional, and cognitive skills” von Bediou und anderen aus dem Jahr 2018. Die Forschenden arbeiten an den Universitäten Genf, Kalifornien Santa Barbara, Wisconsin-Madison und dem Teachers College New York.

Gut zu wissen

Die Übersichtsarbeit, zu der Sie heute eine Zusammenfassung lesen, ist eine Metaanalyse. Forschende, die eine Metaanalyse machen, suchen zuerst die Ergebnisse aller Studien zu einer bestimmten Frage (z. B. “Wie gut hilft eine bestimmte Psychotherapie?”). Dann fassen sie die Ergebnisse dieser Studien zusammen.

Warum macht man das? Eine Metaanalyse hat zwei Ziele:

1. Die Metaanalyse gibt einen Überblick über alle Studien, die diese Frage schon untersucht haben. Sie beschreibt, wer schon zu einer Frage geforscht hat (z. B., wer schon alles diese Psychotherapie untersucht hat). Außerdem steht dort, was in den einzelnen Studien herauskam (z. B., ob und wie gut die Psychotherapie geholfen hat).

2. Die Metaanalyse liefert außerdem einen Wert, der die Ergebnisse aller Studien zusammenfasst. Dafür nehmen die Forschenden die Einzelergebnisse aller gefundenen Studien und berechnen ein Gesamtergebnis. Es ist dabei wichtig, dass die gefundenen Studien zu einem Thema ziemlich ähnlich sind. Wenn sich die Studien sehr unterscheiden, wird das berechnete Gesamtergebnis der Metaanalyse ungenau. Das berechnete Gesamtergebnis liefert den Forschenden die Antwort auf die anfangs gestellte Frage (z. B. “Über alle Studien hinweg hilft die Psychotherapie sehr gut.”). Dieses Gesamtergebnis hat eine viel höhere Aussagekraft als die Ergebnisse der einzelnen Studien.

Was war das Ziel der Übersichtsarbeit?

Hintergrund: Videospiele spielen eine immer größere Rolle im Alltag von Kindern, Jugendlichen und Erwachsenen. Besonders beliebt sind Action-Videospiele. Forschende beschäftigen sich schon lange damit, inwiefern sich solche Spiele positiv oder negativ auf die Entwicklung der Wahrnehmung, der Aufmerksamkeit und des Denkens auswirken.

Forschungsfrage: Mit ihrer Übersichtsarbeit wollten die Forschenden herausfinden: Welchen Einfluss hat das regelmäßige Spielen von Action-Videospielen auf die Wahrnehmung, die Aufmerksamkeit und das Denken?

Kernaussage der Übersichtsarbeit

Das regelmäßige Spielen von Action-Videospielen hat, im Vergleich zum Nicht-Spielen, positive Auswirkungen auf die Wahrnehmung, die Aufmerksamkeit und das räumliche Denken. Es zeigen sich deutliche Unterschiede zwischen Action-Videospielenden und Nicht-Spielenden.

Wie sind die Forschenden vorgegangen?

Welche Studien haben die Forschenden gesucht?
Die Forschenden suchten nach Studien, die die Wahrnehmung, die Aufmerksamkeit und das räumliche Denken von Action-Videospielenden und Nicht-Spielenden miteinander verglichen.

Welche Studien haben die Forschenden gefunden?
Die Forschenden fanden insgesamt 116 Studien aus den Jahren 2000 bis 2015. Aus diesen Studien konnten sie 198 Ergebnisse, zu einer Metaanalyse zusammenfassen.

Was haben die Forschenden gemacht?
In allen 116 Studien betrachteten die Forschenden die Unterschiede des Videospielverhaltens und davon abhängige Unterschiede in der Wahrnehmung, der Aufmerksamkeitslenkung und dem räumlichen Denken.

Was haben die Forschenden untersucht?

- Unterschiede im Videospielverhalten:
  - regelmäßiges Spielen von Action-Videospielen (Action-Videospielende)
  - Spielen anderer oder überhaupt keiner Videospiele (Nicht-Spielende)

- Denken, Aufmerksamkeit und Wahrnehmung
  - Wahrnehmung
  - Aufmerksamkeitslenkung
  - räumliches Denken
  - gleichzeitiges Bearbeiten mehrerer Aufgaben

Was sind die wichtigsten Ergebnisse?

- Über 116 Studien hinweg zeigten Action-Videospielende höhere Wahrnehmungsleistungen als Nicht-Spielende. Die Effektstärke Hedges g betrug hier $g = 0.78$. Das ist ein mittelgroßer Unterschied zwischen Action-Videospielenden und Nicht-Spielenden.

» Auch im Hinblick auf bewusste Aufmerksamkeitslenkung zeigten Action-Videospielende höhere Leistungen als Nicht-Spielende. Die Effektstärke Hedges g betrug hier g = 0.63. Das ist ein mittelgroßer Unterschied zwischen Action-Videospielenden und Nicht-Spielenden.

» Action-Videospielende zeigten höhere Leistungen als Nicht-Spielende beim räumlichen Denken. Die Effektstärke Hedges g betrug hier g = 0.75. Das ist ein mittelgroßer Unterschied zwischen Action-Videospielenden und Nicht-Spielenden.

» Action-Videospielende zeigten höhere Leistungen als Nicht-Spielende bei der gleichzeitigen Bearbeitung mehrerer Aufgaben. Die Effektstärke Hedges g betrug hier g = 0.55. Das ist ein mittelgroßer Unterschied zwischen Action-Videospielenden und Nicht-Spielenden.

Was bedeuten die Ergebnisse im Alltag?

Action-Videospielende haben eine bessere Wahrnehmung und Aufmerksamkeit und ein besseres räumliches Denkvermögen als Nicht-Spielende. Damit kann das Spielen von Action-Videospielen als Training bei Sehstörungen, bei Aufmerksamkeitsproblemen (z.B. Lese-Rechtschreib-Schwäche) oder für die Bewältigung beruflicher Anforderungen (z.B. Fliegen, Leistungssport) hilfreich sein. Hierzu sind jedoch weitere Studien nötig.

Was ist noch zu beachten?

Verzerrung der Ergebnisse durch eingeschränktes Veröffentlichen von Studien

» *Worum geht es?* Eindeutige Forschungsergebnisse lassen sich leichter veröffentlichen als uneindeutige Ergebnisse. Das ist für Übersichtsarbeiten problematisch, weil sie diese unveröffentlichten, uneindeutigen Ergebnisse nicht mitberücksichtigen können.

» *Was bedeutet das für die vorliegende Übersichtsarbeit?* Die Forschenden dieser Übersichtsarbeit fanden Hinweise auf solche unveröffentlichten Studien mit uneindeutigen Ergebnisse. Ob die Unterschiede in der Wahrnehmung, der Aufmerksamkeit und dem räumlichen Denken zwischen Action-Videospielenden und Nicht-Spielenden tatsächlich kleiner oder größer ist als in ihrer Übersichtsarbeit berechnet, bleibt damit unklar.

Wer hat die Studie finanziert?
In der Veröffentlichung der Übersichtsarbeit können keine Angaben dazu gefunden werden, wie die Forschung finanziert wurde.

Gab es Interessenkonflikte?
In der Veröffentlichung der Übersichtsarbeit geben die Forschenden an, dass folgende Interessenkonflikte vorliegen: Die Forschende Daphne Bavelier gibt an Mitglied des Wissenschaftlichen Beirats von Akili Interactive in Boston (einem Biotechnologieunternehmen) zu sein.

Welche Psychotherapien helfen am besten bei einer Depression?

Eine Übersichtsarbeit

Diese Zusammenfassung bezieht sich auf die Übersichtsarbeit mit dem Titel “Comparative efficacy of seven psychotherapeutic interventions for patients with depression: a network meta-analysis” von Barth und anderen aus dem Jahr 2013. Die Forschenden arbeiten an den Universitäten Bern, London und Amsterdam.

Gut zu wissen

Die Übersichtsarbeit, zu der Sie heute eine Zusammenfassung lesen, ist eine Metaanalyse. Forschende, die eine Metaanalyse machen, suchen zuerst die Ergebnisse aller Studien zu einer bestimmten Frage (z. B. “Wie gut hilft eine bestimmte Psychotherapie?”). Dann fassen sie die Ergebnisse dieser Studien zusammen.

Warum macht man das? Eine Metaanalyse hat zwei Ziele:

1. Die Metaanalyse gibt einen Überblick über alle Studien, die diese Frage schon untersucht haben. Sie beschreibt, wer schon zu einer Frage geforscht hat (z. B., wer schon alles diese Psychotherapie untersucht hat). Außerdem steht dort, was in den einzelnen Studien herauskam (z. B., ob und wie gut die Psychotherapie geholfen hat).

2. Die Metaanalyse liefert außerdem einen Wert, der die Ergebnisse aller Studien zusammenfasst. Dafür nehmen die Forschenden die Einzelergebnisse aller gefundenen Studien und berechnen ein Gesamtergebnis. Es ist dabei wichtig, dass die gefundenen Studien zu einem Thema ziemlich ähnlich sind. Wenn sich die Studien sehr unterscheiden, wird das berechnete Gesamtergebnis der Metaanalyse ungenau. Das berechnete Gesamtergebnis liefert den Forschenden die Antwort auf die anfangs gestellte Frage (z. B. “Über alle Studien hinweg hilft die Psychotherapie sehr gut.”). Dieses Gesamtergebnis hat eine viel höhere Aussagekraft als die Ergebnisse der einzelnen Studien.

Was war das Ziel der Übersichtsarbeit?

Hintergrund: Psychotherapien können Menschen mit psychischen Erkrankungen helfen. Das haben schon viele Studien und Übersichtsarbeiten herausgefunden. Es gibt unterschiedliche Arten von Psychotherapien. Bei Menschen mit einer leichten bis mittelschweren Depression helfen diese verschiedenen Therapien möglicherweise unterschiedlich gut.

Forschungsfrage: In dieser Übersichtsarbeit wollten die Forschenden herausfinden: Welche der verschiedenen Arten von Psychotherapien helfen Menschen mit leichten bis mittelschweren Depressionen?

Kernaussage der Übersichtsarbeit

Alle untersuchten Arten von Psychotherapien helfen Menschen mit Depressionen ähnlich gut. Sie helfen alle besser als wenn Menschen mit Depression gar keine Psychotherapie machen.

Wie sind die Forschenden vorgegangen?

Welche Studien haben die Forschenden gesucht?
Die Forschenden suchten nach Studien, die verschiedene Arten von Psychotherapien miteinander verglichen. Dabei sollten die Studien untersuchen, wie gut die unterschiedlichen Arten von Psychotherapien bei erwachsenen Menschen mit Depression helfen.

Welche Studien haben die Forschenden gefunden?
Die Forschenden fanden insgesamt 198 Studien aus den Jahren 1982 bis 2020, deren Ergebnisse sie zu einer Metaanalyse zusammenfassen konnten. Insgesamt sind das Studienergebnisse von 15 118 Menschen mit Depressionen.

Was haben die Forschenden gemacht?
In den 198 Studien schauten die Forschenden, zu welcher Art die jeweils untersuchte Psychotherapie gehört. Sie überprüften dann, wie gut die jeweilige Psychotherapieart half. Die Forschenden untersuchten auch, ob der Behandlungserfolg mit anderen Merkmalen der Menschen mit Depressionen zusammenhing. Zum Beispiel schauten sie, ob eine durch Fachleute abgesicherte Diagnose der Depression vorlag.

Was haben die Forschenden untersucht?

» Verschiedene Arten von Psychotherapie:
  - Kognitive Verhaltenstherapie
  - Nicht-direktive supportive Therapie
  - Verhaltensaktivierung
  - Psychodynamische Psychotherapie
  - Problemlösetherapie
  - Interpersonelle Psychotherapie
  - Training sozialer Fertigkeiten

» Merkmale der untersuchten Menschen mit Depressionen (z. B. Vorliegen einer abgesicherten Diagnose)

» Wie gut die Psychotherapie half: Schwere der Depression nach einer Psychotherapie

Was sind die wichtigsten Ergebnisse?

» Jede einzelne Art der Psychotherapie half Menschen mit Depressionen besser als keine Psychotherapie. Die Effektstärke Cohen’s d betrug über die 198 Studien hinweg d = - 0.62 bis d = - 0.92. Das ist ein mittelgroßer bis großer Unterschied zwischen Menschen mit Depressionen, die keine Psychotherapie machten und denen, die eine Psychotherapie machten.

» Wenn je zwei Arten der Psychotherapie miteinander verglichen wurden, half keine der Psychotherapiearten wesentlich besser als die andere. Cohen’s d betrug hier d = 0.01 bis d = - 0.30. Das ist ein sehr kleiner bis kleiner Unterschied zwischen den Psychotherapiearten.

» Die einzige Psychotherapieart, die im Vergleich besser half als ein andere, war die Interpersonelle Psychotherapie. Sie half etwas besser als die Nicht-direktive supportive Therapie. Cohen’s d betrug hier d = - 0.30. Das ist ein kleiner Unterschied zwischen Interpersoneller Psychotherapie und Nicht-direktiver supportiver Therapie.

» Die Forschenden fanden heraus, dass sich die Merkmale der untersuchten Menschen mit Depressionen nicht darauf auswirkten, wie gut eine Psychotherapie half. Cohen’s d betrug hier d = - 0.11 bis d = 0.08. Das ist ein sehr kleiner bis kleiner Einfluss von Merkmalen der untersuchten Menschen mit Depressionen auf den Behandlungserfolg.

Was bedeuten die Ergebnisse im Alltag?

Jede in dieser Übersichtsarbeit untersuchte Art der Psychotherapie kann zur Behandlung von Depressionen empfohlen werden. Im Vergleich zu keiner Behandlung trägt eine Psychotherapie eher zu einer Verbesserung des Befindens bei.

Was ist noch zu beachten?

Verzerrung der Ergebnisse durch eingeschränktes Veröffentlichen von Studien

» *Worum geht es?* Eindeutige Forschungsergebnisse lassen sich leichter veröffentlichen als uneindeutige Ergebnisse. Das ist für Übersichtsarbeiten problematisch, weil sie diese unveröffentlichten, uneindeutigen Ergebnisse nicht mitberücksichtigen können.

» *Was bedeutet das für die vorliegende Übersichtsarbeit?* Die Forschenden dieser Übersichtsarbeit machen keine Angaben dazu, ob es Hinweise auf solche unveröffentlichten, uneindeutigen Ergebnisse gab. Ob der Behandlungserfolg der Psychotherapien tatsächlich kleiner ist als in dieser Übersichtsarbeit berechnet, bleibt damit unklar.

Wer hat die Studie finanziert?
Die Forschung wurde durch den Schweizerischen Nationalfonds (einen staatlichen Fonds, der Forschung fördert) finanziert.

Gab es Interessenkonflikte?
In der Veröffentlichung der Übersichtsarbeit können keine Angaben dazu gefunden werden, ob ein Interessenkonflikt vorliegt.

Wie wirken sich der Glaube an sich selbst und der Umgang mit Problemen auf das seelische Wohlbefinden aus?

Eine Übersichtsarbeit

Diese Zusammenfassung bezieht sich auf die Übersichtsarbeit mit dem Titel “Coping as a mediator between locus of control, competence beliefs, and mental health: A systematic review and structural equation modelling meta-analysis.” von Groth und anderen aus dem Jahr 2019. Die Forschenden arbeiten an den Universitäten Bern, Heidelberg, Genf, und Düsseldorf.

Gut zu wissen

Die Übersichtsarbeit, zu der Sie heute eine Zusammenfassung lesen, ist eine Metaanalyse. Forschende, die eine Metaanalyse machen, suchen zuerst die Ergebnisse aller Studien zu einer bestimmten Frage (z. B. “Wie gut hilft eine bestimmte Psychotherapie?”). Dann fassen sie die Ergebnisse dieser Studien zusammen.

Warum macht man das? Eine Metaanalyse hat zwei Ziele:

1. Die Metaanalyse gibt einen Überblick über alle Studien, die diese Frage schon untersucht haben. Sie beschreibt, wer schon zu einer Frage geforscht hat (z. B., wer schon alles diese Psychotherapie untersucht hat). Außerdem steht dort, was in den einzelnen Studien herauskam (z. B., ob und wie gut die Psychotherapie geholfen hat).

2. Die Metaanalyse liefert außerdem einen Wert, der die Ergebnisse aller Studien zusammenfasst. Dafür nehmen die Forschenden die Einzelergebnisse aller gefundenen Studien und berechnen ein Gesamtergebnis. Es ist dabei wichtig, dass die gefundenen Studien zu einem Thema ziemlich ähnlich sind. Wenn sich die Studien sehr unterscheiden, wird das berechnete Gesamtergebnis der Metaanalyse ungenau. Das berechnete Gesamtergebnis liefert den Forschenden die Antwort auf die anfangs gestellte Frage (z. B. “Über alle Studien hinweg hilft die Psychotherapie sehr gut.”). Dieses Gesamtergebnis hat eine viel höhere Aussagekraft als die Ergebnisse der einzelnen Studien.

Was war das Ziel der Übersichtsarbeit?

Hintergrund: Verschiedene Faktoren beeinflussen das seelische Wohlbefinden. Zum Beispiel unterscheiden sich Menschen darin, wie sie mit Problemen umgehen. Sie unterscheiden sich auch darin, wie sehr sie an sich selbst glauben. Beides kann das seelische Wohlbefinden beeinflussen.

Forschungsfrage: In ihrer Übersichtsarbeit wollten die Forschenden herausfinden:

1. Wirkt sich der Glaube an sich selbst darauf aus, wie man mit Problemen umgeht – und verändert sich deswegen das seelische Wohlbefinden?

2. Oder wirkt sich die Art und Weise, wie man mit Problemen umgeht, darauf aus, wie sehr man an sich selbst glaubt – und verändert sich deswegen das seelische Wohlbefinden?

Kernaussage der Übersichtsarbeit

Die Übersichtsarbeit zeigt: Der Glaube an sich selbst hängt unter bestimmten Umständen mit seelischem Wohlbefinden zusammen. Wer glaubt, positive Ereignisse lägen außerhalb der eigenen Kontrolle, der könnte langfristig ein niedrigeres seelisches Wohlbefinden haben.

Wie sind die Forschenden vorgegangen?

Welche Studien haben die Forschenden gesucht?
Sie suchten Studien zum Zusammenhang zwischen seelischem Wohlbefinden und dem Glauben an sich selbst. Der Glaube an sich selbst war aufgeteilt in: Denkweisen über die eigenen Fähigkeiten sowie Denkweisen über die eigene Kontrolle über Ereignisse. Die Studien mussten außerdem untersuchen, welche Rolle die Art und Weise, wie wir mit Problemen umgehen, dabei spielen.

Welche Studien haben die Forschenden gefunden?
Sie fanden 15 Studien aus den Jahren 1996 bis 2016, deren Ergebnisse sie zu einer Metaanalyse zusammenfassen konnten. Insgesamt sind das Studienergebnisse von 3 986 Menschen.

Was haben die Forschenden gemacht?
In den 15 Studien schauten sie sich verschiedene Kombinationen an, wie sich der Glaube an sich selbst und die Art und Weise, wie wir mit Problemen umgehen, auf das seelische Wohlbefinden auswirkt.

Was haben die Forschenden untersucht?

- Faktoren, die sich auf das seelische Wohlbefinden auswirken könnten:
  - Glaube an sich selbst
    - Denkweise über die eigenen Fähigkeiten
      - günstig (z.B. hohes Selbstbewusstsein, Zufriedenheit mit den eigenen Fähigkeiten)
      - ungünstig (z.B. niedriges Selbstbewusstsein, Unzufriedenheitmit den eigenen Fähigkeiten)
    - Denkweise über die eigene Kontrolle über Ereignisse
      - günstig (eigene Kontrolle über positive Ereignisse)
      - ungünstig (keine eigene Kontrolle über positive Ereignisse)
  - Art und Weise, wie die untersuchten Personen mit Problemen umgehen
      - günstig (z .B. aktive Auseinandersetzung mit der Situation)
      - ungünstig (z.B. Verdrängung, Substanzmissbrauch)

- seelisches Wohlbefinden
    - niedrig bis hoch

Was sind die wichtigsten Ergebnisse?

- Wenn Personen glaubten, dass sie über negative, aber nicht über positive Ereignisse Kontrolle haben, gingen sie langfristig eher ungünstig mit Problemen um. Dies führte dann zu schlechterem seelischen Wohlbefinden. Die Effektstärke Beta (ß) betrug über die 15 Studien hinweg ß = 0.05. Dies ist ein kleiner Zusammenhang.

- Es gab im Hinblick auf das seelische Wohlbefinden keine anderen bedeutsamen Zusammenhänge zwischen dem Glauben an sich selbst und der Art und Weise, wie die Personen mit Problemen umgingen.

Was bedeuten die Ergebnisse im Alltag?

Die Ergebnisse zeigen, dass sich ungünstige Denkweisen bezüglich der eigenen Kontrolle von Ereignissen schlecht auf den Umgang mit Problemen und damit schlecht auf das seelische Wohlbefinden auswirken. Für Maßnahmen zur Verbesserung des seelischen Wohlbefindens empfehlen die Forschenden daher: Zuerst sollten Übungen gemacht werden, die diese ungünstigen Denkweisen vermindern. Erst danach sollten Übungen gemacht werden, die den Umgang mit den Problemen an sich verbessern.

Was ist noch zu beachten?

Verzerrung der Ergebnisse durch eingeschränktes Veröffentlichen von Studien

- *Worum geht es?* Eindeutige Forschungsergebnisse lassen sich leichter veröffentlichen als uneindeutige Ergebnisse. Das ist für Übersichtsarbeiten problematisch, weil sie diese unveröffentlichten, uneindeutigen Ergebnisse nicht mitberücksichtigen können.

- *Was bedeutet das für die vorliegende Übersichtsarbeit?* Die Forschenden dieser Übersichtsarbeit machen keine Angaben dazu, ob es Hinweise auf solche unveröffentlichten, uneindeutigen Ergebnisse gab. Ob der Zusammenhang zwischen den untersuchten Faktoren tatsächlich kleiner ist als in dieser Übersichtsarbeit berechnet, bleibt damit unklar.

Wer hat die Studie finanziert?
Die Forschung wurde nicht durch Dritte (z.B. Stiftungen oder Unternehmen) finanziell gefördert oder unterstützt.

Gab es Interessenkonflikte?
In der Veröffentlichung der Übersichtsarbeit können keine Angaben dazu gefunden werden, ob ein Interessenkonflikt vorliegt.

KLARtext 1: Action Video Games

Does playing action video games affect perception, attention, and thinking? A review article

This summary refers to the review article titled “Meta-analysis of action video game impact on perceptual, attentional, and cognitive skills” by Bediou and colleagues from 2018. The researchers work at the University of Geneva, the University of California, Santa Barbara, the University of Wisconsin-Madison, and Teachers College, New York.

Good to know:

The review article you are reading a summary of today is a meta-analysis. Researchers who conduct a meta-analysis first look for the results of all studies on a certain question (e.g., “How well does a certain psychotherapy help?”). Then they summarize the results of these studies.

Why are they doing that? A meta-analysis has two goals:

1) The meta-analysis provides an overview of all studies that have already investigated this question. It describes who researched a question (e.g., who investigated this psychotherapy). Moreover, it summarizes the findings from the individual studies (e.g., whether and to what extent the psychotherapy was effective).
2) The meta-analysis gives a value for the combined results of all studies found. To do this, the researchers take the individual results of all studies and calculate an overall result. It is important that the studies on a certain question are quite similar. If the studies differ a lot, the calculated overall result will be inaccurate. The calculated overall result provides an answer to the initial question (e.g., “Across all studies, the psychotherapy helps very well”). This overall result is more meaningful than the results of individual studies.

What was the goal of the review article?

Background: Video games are becoming more and more important in the everyday lives of children, adolescents, and adults. Action video games are especially popular. Researchers have long studied whether these games have a positive or negative impact on the development of perception, attention, and thinking.

Research question: In their review article, the researchers wanted to find out: What is the influence of regularly playing action video games on perception, attention, and thinking?

Key message of the review article

Regularly playing action video games has a positive effect on perception, attention, and spatial thinking compared to not playing. There are clear differences between action video game players and non-players.

How did the researchers proceed?

What studies did the researchers look for? The researchers looked for studies that compared the perception, attention, and spatial thinking of action video game players and non-players.

Which studies did the researchers find? In total, the researchers found 116 studies published between 2000 and 2015. From these studies, they were able to include 198 results in the meta-analysis.

What did the researchers do? Across the 116 studies, the researchers examined the differences in video gaming behavior and related differences in perception, attention, and spatial thinking.

What did the researchers investigate?

- Differences in video gaming behavior
    - Regular playing of action video games (action video game players)
    - Playing other video games or no video games at all (non-players)
- Thinking, attention, and perception
    - Perception
    - Attention control
    - Spatial thinking
    - Multi-tasking

What are the most important results?

- Across 116 studies, action video game players showed better performance in perception than non-players. The effect size Hedges' g was $g = 0.78$. This is a medium-sized difference between action video game players and non-players.
- Likewise, action video game players demonstrated better attention control than non-players. The effect size Hedges' g was $g = 0.63$. This is a medium-sized difference between action video game players and non-players.

- Action video game players outperformed non-players in spatial thinking. The effect size Hedges' g was $g = 0.75$. This is a medium-sized difference between action video game players and non-players.
- Action video game players were better at multi-tasking than non-players. The effect size Hedges g was $g = 0.55$. This is a medium-sized difference between action video game players and non-players.

What do the results mean for everyday life?

Action video game players have better perception, attention, and spatial thinking than non-players. Hence, playing action video games may be helpful as a form of training for people with visual impairments, attention problems (e.g., dyslexia), or for coping with professional requirements (e.g., flying, professional sports). However, further research is needed in this area.

What else is there to consider?

Distorted results due to the limited publication of studies

- **What is this about?** Clear research results are more likely to be published than unclear results. This creates a problem for review articles because these unpublished, unclear results cannot be included.
- **What does that mean for the present review article?** The researchers found indications of unpublished studies with unclear results. Whether the differences in perception, attention, and spatial thinking between action video game players and non-players were in fact smaller or larger than calculated remains unknown.

Funding of the study: The review article provides no information about the study's funding.

Conflicts of interest: In the review article, the researchers mention that there is a conflict of interest: the researcher Daphne Bavelier states that she is a member of the Scientific Advisory Board of Akili Interactive in Boston (a biotechnology company).

KLARtext 2: Mental Well-being

How do believing in oneself and coping with problems impact mental well-being? A review article

This summary refers to the review article titled “Coping as a mediator between locus of control, competence beliefs, and mental health: A systematic review and structural equation modelling meta-analysis” by Groth and colleagues from 2019. The researchers work at the universities of Bern, Heidelberg, Geneva, and Düsseldorf.

Good to know:

The review article you are reading a summary of today is a meta-analysis. Researchers who conduct a meta-analysis first look for the results of all studies on a certain question (e.g., “How well does a certain psychotherapy help?”). Then they summarize the results of these studies.

Why are they doing that? A meta-analysis has two goals:

1) The meta-analysis provides an overview of all studies that have already investigated this question. It describes who researched a question (e.g., who investigated this psychotherapy). Moreover, it summarizes the findings from the individual studies (e.g., whether and to what extent the psychotherapy was effective).
2) The meta-analysis gives a value for the combined results of all studies found. To do this, the researchers take the individual results of all studies and calculate an overall result. It is important that the studies on a certain question are quite similar. If the studies differ a lot, the calculated overall result will be inaccurate. The calculated overall result provides an answer to the initial question (e.g., “Across all studies, the psychotherapy helps very well”). This overall result is more meaningful than the results of individual studies.
3)

What was the goal of the review article?

Background: Several factors influence mental well-being. For example, people differ in how they cope with problems. They also differ in how much they believe in themselves. Both can affect their mental well-being.

Research questions: In their review article, the researchers wanted to find out: (1) Does believing in oneself have an impact on how someone copes with their problems – and does this change mental well-being? (2) Or does the way someone copes with their problems have an impact on how they believe in themselves – and does this change mental well-being?

Key message of the review article

The review article shows that, under certain circumstances, believing in oneself is related to mental well-being. People who believe that positive events are beyond their control may experience lower levels of mental well-being in the long term.

How did the researchers proceed?

What studies did the researchers look for? They looked for studies that investigated the relationship between mental well-being and believing in oneself. Believing in oneself was divided into: thinking about one's abilities as well as thinking about one's control over events. Moreover, the studies were required to examine the role that the way individuals cope with their problems plays in this regard.

Which studies did the researchers find? They found 15 studies published between 1996 and 2016, the results of which they summarized in a meta-analysis. In total, these results are based on 3 986 people.

What did the researchers do? In the 15 studies, the researchers were interested in different combinations of how believing in oneself and the way we cope with problems affect mental well-being.

What did the researchers investigate?

- Factors that affect mental well-being
    - Believing in oneself
        - Thinking about one's abilities
            - Favorable (e.g., high self-esteem, satisfaction with one's abilities)
            - Unfavorable (e.g., low self-esteem, dissatisfaction with one's abilities)
        - Thinking about one's control over events
            - Favorable (perceived control over positive events)
            - Unfavorable (no perceived control over positive events)
    - How people cope with problems
            - Favorable (e.g., active engagement with the situation)

        - Unfavorable (e.g., avoidance, substance abuse)
- Mental well-being
        - Low to high

What are the most important results?

- If people believed they had control over negative but not positive events, they tended to cope with problems unfavorably in the long term. This, in turn, led to lower mental well-being. Across the 15 studies, the effect size Beta (ß) was *ß* = 0.05. This indicates a small relationship.
- In terms of mental well-being, there were no other meaningful relationships between believing in oneself and the way people coped with problems.

What do the results mean for everyday life?

The results show that unfavorable thinking, in terms of one's control over events, negatively affects how people cope with problems. Ultimately, this harms mental well-being. Thus, for interventions to improve mental well-being, the researchers firstly recommend exercises to reduce these unfavorable thinking patterns. Only then should exercises be introduced to help people improve the way they cope with the problems.

What else is there to consider?

Distorted results due to the limited publication of studies

- **What is this about?** Clear research results are more likely to be published than unclear results. This creates a problem for review articles because these unpublished, unclear results cannot be included.
- **What does that mean for the present review article?** The researchers of the present review article do not mention whether there were indications of such unpublished, unclear results. Whether the relationship between the factors investigated is in fact smaller than calculated in the review article remains unknown.

Funding of the study: The review article was not financially funded or supported by third parties (e.g., foundations or companies).

Conflicts of interest: In the present review article, no information is provided on whether a conflict of interest exists.

KLARtext 3: Psychotherapy

Which psychotherapies help best against depression? A review article

This summary refers to the review article titled “Comparative efficacy of seven psychotherapeutic interventions for patients with depression: a network meta-analysis” by Barth and colleagues from 2013. The researchers work at the universities of Bern, London, and Amsterdam.

Good to know:

The review article you are reading a summary of today is a meta-analysis. Researchers who conduct a meta-analysis first look for the results of all studies on a certain question (e.g., “How well does a certain psychotherapy help?”). Then they summarize the results of these studies.

Why are they doing that? A meta-analysis has two goals:

1) The meta-analysis provides an overview of all studies that have already investigated this question. It describes who researched a question (e.g., who investigated this psychotherapy). Moreover, it summarizes the findings from the individual studies (e.g., whether and to what extent the psychotherapy was effective).
2) The meta-analysis gives a value for the combined results of all studies found. To do this, the researchers take the individual results of all studies and calculate an overall result. It is important that the studies on a certain question are quite similar. If the studies differ a lot, the calculated overall result will be inaccurate. The calculated overall result provides an answer to the initial question (e.g., “Across all studies, the psychotherapy helps very well”). This overall result is more meaningful than the results of individual studies.

What was the goal of the review article?

Background: Psychotherapy can help people with psychological illnesses. That has been found by many studies and review articles. There are different types of psychotherapies. For people with mild to medium depression, these types of psychotherapies might help to different extents.

Research question: In their review article, the researchers wanted to find out: Which types of psychotherapy help people with mild to medium depression?

Key message of the review article

All types of psychotherapy studied help people with depression to a similar extent. They all help people with depression more than receiving no psychotherapy at all.

How did the researchers proceed?

What studies did the researchers look for? The researchers looked for studies that compared different types of psychotherapy. The studies should investigate how well the different types of psychotherapy help adults with depression.

Which studies did the researchers find? The researchers found 198 studies published between 1982 and 2020, the results of which they summarized in a meta-analysis. In total, these are study results of 15 118 people with depression.

What did the researchers do? In the 198 studies, the researchers examined the type of psychotherapy to which the investigated treatment belonged. Then, they assessed how well the type of psychotherapy helped. The researchers also examined whether the success of the treatment was related to other characteristics of people with depression. For example, they looked at whether professionals had confirmed a diagnosis of depression.

What did the researchers investigate?

- Different types of psychotherapy
    - Cognitive-behavioral therapy
    - Supportive counselling
    - Behavioral activation
    - Psychodynamic psychotherapy
    - Problem-solving therapy
    - Interpersonal psychotherapy
    - Social skills training
- Characteristics of the investigated people with depression (e.g., diagnosis confirmed by a professional)
- How well the psychotherapy helped: Severity of depression after receiving psychotherapy

What are the most important results?

- Each type of psychotherapy helped people with depression better than no psychotherapy. Across the 198 studies, the effect size Cohen's d ranged from *d* = -0.62 to *d* = -0.92. This is a medium to large difference between people with depression who received psychotherapy and those with depression who did not receive psychotherapy.
- When different types of psychotherapy were compared pairwise, no type helped meaningfully more than the others. Cohen's d was from *d* = 0.01 to *d* = -0.30. This is a very small to small difference between the types of psychotherapy.
- The only type of psychotherapy that helped more compared to others was interpersonal psychotherapy. It helped slightly more than supportive counselling. Cohen's d was *d* = -0.30. That is a small difference between interpersonal psychotherapy and supportive counselling.
- The researchers found that the characteristics of the people with depression studied were not related to how much the psychotherapy helped. Cohen's d was from *d* = -0.11 to *d* = 0.08. That is a very small to small influence of the characteristics of the people with depression studied on the success of the treatment.

What do the results mean for everyday life?

Each type of psychotherapy studied in the present review article can be recommended for the treatment of depression. Compared to receiving no treatment at all, psychotherapy is more likely to contribute to an improvement in well-being.

What else is there to consider?

Distorted results due to the limited publication of studies

- **What is this about?** Clear research results are more likely to be published than unclear results. This creates a problem for review articles because these unpublished, unclear results cannot be included.
- **What does that mean for the present review article?** The researchers of the present review article do not state whether there were any indications of such unpublished or unclear results. Thus, whether the success of psychotherapies is in fact smaller than calculated in the review article remains unknown.

Funding of the study: The research has been financially supported by the Swiss National Foundation (a state fund that supports research).

Conflicts of interest: In the review article, no information regarding conflicts of interest is provided.
