# Supplementary material for: “A very first clue on the subject”: A focus group study on users’ perspectives on German plain language summaries of psychological meta-analyses
Source: PLoS One. 2026 Mar 10;21(3):e0343625. doi: 10.1371/journal.pone.0343625 (PMC12974800; doi:10.1371/journal.pone.0343625)
Supplement: S2 File — (PDF) [file pone.0343625.s004.pdf]

S4_File

Interview guide

1. **Warm-up – Psychological information in general**

    - What psychological topics or information are you particularly interested in?
    - What sources do you use to gather information?
    - What experiences have you had with information on psychological topics so far? Are you satisfied with the mentioned offerings?

Stimulus – Three PLSs

2. **First impression**

    - What is your first impression? What did you notice?
    - What do you like about the PLSs?
        - Why? Specific example from the text?
    - What don't you like as much?
        - Why? Specific example from the text?

3. **Aims and benefits from the users' perspective**

    - Why/For what aim would you read such a PLS?
    - What consequences does reading the text have for you? What conclusion do you draw from the text?
    - In what ways could such summaries of psychological research be helpful for you?
    - When would you read PLSs?

4. **Characteristics and criteria from the users' perspective**

    - How should the text be designed and presented to be useful for you?
    - What characteristics should the text not have?
    - To what extent do the PLSs meet these characteristics?
        - Specific example from the text?
    - Which of these characteristics are not fulfilled by the PLSs?
        - Specific example from the text?

5. **Closure**

    - Would you recommend this kind of PLS? If yes, to whom?
    - Is there anything we haven't discussed that, from your perspective, would be important?
