# Supplementary material for: “A very first clue on the subject”: A focus group study on users’ perspectives on German plain language summaries of psychological meta-analyses
Source: PLoS One. 2026 Mar 10;21(3):e0343625. doi: 10.1371/journal.pone.0343625 (PMC12974800; doi:10.1371/journal.pone.0343625)
Supplement: Attachment — Submitted filename: Rebuttal Letter.docx [file pone.0343625.s007.docx]

**Reviewer Comments:**

**All comments of the reviewers were answered point-by point below. Given page numbers and sections, refer to the version with track changes.**

| **Reviewer #3** | |
| --- | --- |
| **First of all, we thank the reviewer for the critical reading of our manuscript and the constructive and very helpful remarks.** | |
| **Reviewer Comments** | **Response** |
| **Abstract** | |
| **Mentions general population, then laypeople. Inconsistent terminology.** | In the revised version of the abstract, we have replaced the term “general population” with “lay audiences” (p.2, l.28). |
| Abstract - “We wanted to know what the aims and benefits of reading psychological PLSs for them are and how these PLSs should be designed, in their point of view, in order to be useful.” – poor grammar. | **We have changed the sentence into: “We wanted to understand the aims and benefits of reading psychological PLSs from their perspective, and how these PLSs should be designed to be useful to them.” (p.2, l.31-33)** |
| Abstract - Mixed tense. | **In the revised version of the abstract, we now avoid mixed tenses.** |
| Abstract - This abstract does not give me a good understanding of the manuscript, nor is it the best representation of the study. I would recommend re-writing it. | **We appreciate the reviewer’s comment. We have rewritten the abstract to provide a better understanding of the manuscript.** |
| **Introduction** | |
| Need reference for study mentioned lines 57-59. Assume it is reference 2, but need to include for both sentences about this study. | We cite reference 2 in both sentences now (p.3, l.67) |
| I don’t understand the use of reference number 5 and the example of psychology and climate change in the context of communicating to the public – after reading this paper (reference 5), it does not seem to hold appeal for a broad audience | The reference number 5 is cited for the statement that “psychological evidence becomes increasingly important (...) to deal with problems like climate change that affect society as a whole.” Here, we do not want to make the point that the findings currently are communicated to the public (which they are often not) but that they provide solutions for problems that affect all of us and therefore should be shared with society as a whole in the future, and not only with scientists. We rephrased the sentence so that our point becomes clearer (p.3, l.71-74). |
| Line 74 – need to clarify that you are discussing text-based PLSs in the opening sentence. | We have changed the sentence to point out that PLSs are text-based: “Text-based Plain Language Summaries (PLSs) represent one approach for communicating research findings to the public.” (p.4, l.84-85) |
| Line 82 – Autism is not a discipline – it is a topic within the field of health and medicine | Thank you for this advice. We have replaced “disciplines“ with „fields and topics“ (p.4, l. 91-92) |
| Line 84-86 – This sentence needs a reference “This is especially challenging for psychological research as the jargon of psychological researchers often sounds like plain language, but is actually a technical term (e.g., “motivation”).” Motivation should not be in quotation marks within the brackets. | We added a reference for this sentence (p.4, l. 97). |
| Line 95 – this is poor grammar – “The leading question for the project was “How should a good PLS of a psychological meta-analysis look like?” | Thank you for pointing this out. We have improved the grammar of this sentence (p. 5, l. 106). |
| The authors need to keep referencing their previous work throughout the paragraph, after referencing it on line 104. | Reference 8 is cited in all relevant sentences now (p.5, l. 116, 120, 122). |
| Referring to comment 1.3 by reviewer 1, more details is need on what it is that makes psychological research methods distinct? | We have added a more detailed explanation: “A separate investigation of psychological PLSs is essential for at least two reasons: first, the distinctness of psychological research: as the subject is to examine the mind, the constructs of interest are often not directly observable, but must be inferred or operationalized by manipulating proxy variables. Furthermore, studies often rely on correlation designs and context-sensitive interpretations. Thus, psychology is a very nuanced and complex field with a growing complexity of research methods. Since it is a relatively new field, methods and subdisciplines are evolving rapidly. And second, the specificity of psychological jargon, in that the technical terms seemingly overlap with plain language.” (p.6, l. 134-142) |
| **Methods** | |
| Line 184 - Why are the authors using reference 27 and mentioning the use of focus groups in the context of market research, when the context for this study is scientific research | Thank you for pointing this out. The respective sentence has been removed. |
| Did someone from the research team check the PLSs to ensure they were written according to the guidelines provided before they were used in the interviews? | As is written in the text, the “PLSs were written (...) based on the criteria of the first version of our own PLS guideline”. They were written by the study authors themselves, which was not mentioned in the text. We added this information to clarify in the text: “All PLSs were written in German based on the criteria of the first version of the PLS guideline [28] by AC, MK, MS and GB, who are employees of the Leibniz Institute for Psychology and who have experience with writing these kind of PLSs.” (p.8, l.208-11) |
| It would be useful to understand the participant selection process more fully to ensure there was no bias i.e., how were participants selected from the pool of respondents i.e., how they aimed “to maximize variation of socio-demographic characteristics (age, sex, education level) in the sample”. Were group numbers for each cohort determined prior to recruitment? | We describe the recruitment and selection process more clearly now:   1. We have stated that the number of groups was determined before recruitment started: “We aimed to conduct two focus group interviews with laypeople, one focus group interview with science communicators, and one with psychologists working as counselor and/or psychotherapists (“psychological practitioners”) (p.8, l. 202-04) 2. We have provided more details on the recruitment process in S5 table. 3. We have explained why we had to select participants who fulfilled the inclusion criteria: “For laypeople, expressions of interest exceeded the number of available spots, so one researcher (CB) selected participants with the aim of maximizing variation in socio-demographic characteristics (age, sex, education level) within this group”. (p.10, l.240-43) |
| Line 192 – I would have liked to review the three PLSs that were part of the interviews, however S3 file was only provided in German. I am fluent in English only. | We have now provided an English translation of the three PLS in S4 File. Please note that this is a translated version of the original PLSs provided for informational purposes only. Due to the nature of plain language summaries, the exact content and complexity (compared to scientific language) of the original cannot be fully reproduced in translation. |
| Line 245 - Was there a reason these topics were chosen for the three PLSs? Given that it is stated that each focus group session ended with participants being asked about their willingness to recommend “this type of PLS”, I am wondering if the PLSs were chosen for a specific reason. Also, what do the authors mean by “type” of PLS? Format, length, topic etc. | Thank you for pointing this out. Prior to this question at the end, the moderator had explained that the group discussion was about PLSs of psychological meta-analyses and that the PLSs presented were prototypes. Moreover, a website with many PLSs on different psychological topics will be developed subsequently. The question „would you recommend this kind of PLS?“ referred to those psychological PLSs in the form which they had been presented. The topics were chosen so that they cover different areas of psychological research (clinical research, general psychology, mental health / individual differences). |
| Line 255 - It would be useful for the authors to include their rationale for their method of data analysis based on reference 29. Also, a short description of this method would be helpful as many readers might not be familiar with it. | We now provide a rationale for choosing the approach according to Kuckartz and Rädiker and have explained the method using our analysis example (p.13, l. 288-300) |
| **Results** | |
| Line 273 – I would like to see a summary of the key results, not details about the focus groups, some of which belongs in the Methods section. Authors being with 3.1. User’s aims without any introduction. | Thank you for this suggestion. We have integrated the introductory paragraph into section 2.2 Setting and participants (p.10, l.245-49). |
| Table 1 – Participant characteristics. I suggest the authors remember that this paper is likely to appeal to an international audience, so providing context for education levels would be helpful. | To provide context without going beyond the scope, we have classified educational levels now as low, moderate and high, and summarised the categories qualification for university and university degree in Table 1. |
| My concern is that interviews were constrained by the framework the authors developed and reported in a previous systematic review (reference 8). | Thank you for pointing this out. For our analysis, we chose a deductive-inductive categorization process according to Kuckartz and Rädiker. This means we based the analysis on a previously developed categorisation system, but allowed additional categories and subcategories to be derived from our data. In other words, we used the framework from Stoll et al. only as a starting point, but inductive building of main and subcategories was possible and was done for subcategories. Moreover, our interview guide contained only open questions on the aims and characteristics of PLSs. |
| Line 502 – what are the authors reporting on aspects of the PLSs that the participants “missed”? This does not appear to be in alignment with the study aims as this is an evaluation of the ability of the participants to understand the information in the PLS. No criteria for this type of rating was established in the Methods. | Thank you for your comment. Our second research question was „How should PLSs, from the users’s point of view, be designed in order to be useful?“ We explained at the beginning of each discussion that the PLSs presented during the discussion are text-based summaries of psychological research. During the interviews we explicitly asked participants which characteristics they find important for psychological PLSs from their point of view. We only analysed participants‘ comments and did not assess their ability to understand the information. We have included a participant’s quote to support the statement (p.27, l.570-71).  When revisiting this paragraph we realized that there was some ambiguity here and that our sentence might have been misleading. We, thus, clarified the sentence so that it becomes clearer what we mean: “Participants of all groups pointed out that a general statement or ranking about reliability of results, which would help them to assess the meaningfulness and trustworthiness of the results, was lacking.” (p.7, l. 567- 69) |
| **Discussion** | |
| Line 637 – how is psychological research different to medical research? Isn’t psychological research a sub-set of medical research? | Psychological research is not a sub-discipline of medical research but a discipline in its own right. The subject is human experience, cognitions and behaviour. There are some overlaps between psychiatric medical research and research in clinical psychology when pathological behaviour is investigated - but clinical psychology is only one out of many sub-disciplines of psychology. |
| Line 644 – this should be under the sub-heading of Strengths and Weaknesses | Thank you for pointing this out. The aspect of representativeness is already mentioned under 4.3 Strengths and Limitations, so we have removed it from 4.2 Main findings in Context. |
| Line 678 – I do not think the authors can state that the study sample was diverse, nor that diversity of the sample was a strength of the study, since participants were all recruited from Germany. Authors took steps to create diversity, however, their claim of diversity is overstated. Beginning line 701, authors outline the limited diversity of the sample as a study limitation, so there is clear confusion about this issue. | Thank you for your comment. We removed the sentence „A strength of this study is the diversity of our sample.“ from section 4.3 Strengths and Limitations to avoid confusion. However, it is important for us to emphasize that in this case it does not make sense to relate diversity to the place of residence of the participants. The target group of the texts are persons who can speak German as the texts are in German. Interestingly, one participant explicitly appreciated the fact that the information was provided in their native language rather than in English during the interviews. |
| Lines 684 – 687 – the first sentence is non-sensical and the following sentence is not a particular strength as it is standard practice for qualitative research. | We removed both sentences from section 4.3 |
| Line 692 – authors mention saturation for the first time. This should have been addressed in the Methods. | We have addressed saturation in the methods (p.8, l. 206). |
| Line 697 – If KLARpsy did not exist at the time of the study, why is it mentioned in line 97 of the Introduction? Authors should correct this inconsistency. | Thank you for pointing this out. We have removed the sentence in line 107 to avoid confusion. However, the development of KLARpsy is still mentioned in section 4.2 Main findings in context (p. 35, l. 698). |
| Line 707 – This statement is unsupported - “For example, it is possible that people with a lower level of education did not feel motivated to participate in the study”. | We reformulated this sentence so that it is clear that this is only one possible hypothetical explanation: “For example, there is a possibility that it is mainly higher educated people who are interested in taking part in psychological studies on psychological plain language summaries – and that it is also these people who are more likely to read psychological PLSs.” (p.37, l.758) |
| **Additional authors‘ comments** | |
| In the last peer review, we mentioned that „we have only saved the durations, but not which duration corresponds  to which focus group. The recordings have already been deleted in the meantime. Only a  range and the average can be provided”. Nevertheless, we missed to delete durations from table 1 in the submitted manuscript, so we removed the corresponding line in the current version of Table 1.The range and the average have still been reported in line 255-56. | |

**Reviewer #4**

**We thank the reviewer for the critical reading of our manuscript.**
