# Supplementary material for: “A very first clue on the subject”: A focus group study on users’ perspectives on German plain language summaries of psychological meta-analyses
Source: PLoS One. 2026 Mar 10;21(3):e0343625. doi: 10.1371/journal.pone.0343625 (PMC12974800; doi:10.1371/journal.pone.0343625)
Supplement: Attachment — Submitted filename: Reviewer Comments to revision.docx [file pone.0343625.s008.docx]

**Item 1**

Reviewer comment – revision 1
I don’t understand the use of reference number 5 and the example of psychology and climate change in the context of communicating to the public – after reading this paper (reference 5), it does not seem to hold appeal for a broad audience

Author response
The reference number 5 is cited for the statement that “psychological evidence becomes increasingly important (...) to deal with problems like climate change that affect society as a whole.” Here, we do not want to make the point that the findings currently are communicated to the public (which they are often not) but that they provide solutions for problems that affect all of us and therefore should be shared with society as a whole in the future, and not only with scientists. We rephrased the sentence so that our point becomes clearer (p.3, l.71-74).

Reviewer comment – revision 2
I appreciate the authors’ response, however, it does not address my comment, and the sentence is still not clear. Perhaps the authors could expand on how psychological findings help society deal with problems such as climate change.

Authors' response

We reviewed this paragraph and reformulated it so that it hopefully becomes clearer. Also, we added another reference of the American Psychological Association on the interface between psychology and global climate change (p. 3, l. 63-70).

Reviewer comment – revision 3

Thank you, however, please remember to reference every statement made.

**Item 2**

Reviewer comment – revision 1
Referring to comment 1.3 by reviewer 1, more details is need on what it is that makes psychological research methods distinct?

Author response
We have added a more detailed explanation: “A separate investigation of psychological PLSs is essential for at least two reasons: first, the distinctness of psychological research: as the subject is to examine the mind, the constructs of interest are often not directly observable, but must be inferred or operationalized by manipulating proxy variables. Furthermore, studies often rely on correlation designs and context-sensitive interpretations. Thus, psychology is a very nuanced and complex field with a growing complexity of research methods. Since it is a relatively new field, methods and subdisciplines are evolving rapidly. And second, the specificity of psychological jargon, in that the technical terms seemingly overlap with plain language.” (p.6, l. 134-142)

Reviewer comment – revision 2

I appreciate this detailed response. A minor note – authors state that there are two reasons, however I have located four separate points, all valid in their own right. For example:
1. the distinctness of psychological research: as the subject is to examine the mind, the constructs of interest are often not directly observable, but must be inferred or operationalized by manipulating proxy variables.
2. Furthermore, studies often rely on correlation designs and context-sensitive interpretations
3. psychology is a very nuanced and complex field with a growing complexity of research methods. Since it is a relatively new field, methods and subdisciplines are evolving rapidly.
4. the specificity of psychological jargon, in that the technical terms seemingly overlap with plain language

Re-writing this paragraph for clarity would be helpful. It should also be referenced throughout.

Authors' response

Thank you for your feedback. We re-wrote the paragraph to improve clarity and added references (p. 5, l. 127-142).

Reviewer comment – revision 3

I note the revisions to this section, however it still lacks clarity and contains jargon or technical terms not explained to the reader. The introduction of the Big Five Model of personality is poorly explained, as is the “recent phenomenon that has come to light is the overuse of psychological technical terms in everyday language, which results in the expansion of their meaning”. Examples of these technical terms would strengthen the argument better than the introduction of the Big Five Model. It should be noted however, that technical terms and jargon is not unique to the field of psychology, as the authors seem to imply in lines 133-134.

**Item 3**

Reviewer comment – revision 1
It would be useful to understand the participant selection process more fully to ensure there was no bias i.e., how were participants selected from the pool of respondents i.e., how they aimed “to maximize variation of socio-demographic characteristics (age, sex, education level) in the sample”. Were group numbers for each cohort determined prior to recruitment?

Author response
We describe the recruitment and selection process more clearly now:
1. We have stated that the number of groups was determined before recruitment started: “We aimed to conduct two focus group interviews with laypeople, one focus group interview with science communicators, and one with psychologists working as counsellor and/or psychotherapists (“psychological practitioners”) (p.8, l. 202-04)
2. We have provided more details on the recruitment process in S5 table.
3. We have explained why we had to select participants who fulfilled the inclusion criteria: “For laypeople, expressions of interest exceeded the number of available spots, so one researcher (CB) selected participants with the aim of maximizing variation in socio-demographic characteristics (age, sex, education level) within this group”. (p.10, l.240-43)


Reviewer comment – revision 2
1. Addressed with thanks.
2. Thank for the additional detail regarding recruitment. I note on p9 authors state “The detailed recruitment strategy is reported in S5 Table.” I find the level of information in this table is lacking in detail. I do not understand why this information was removed from the Methods section and put in a supplementary table. It could easily be summarised in a few sentences in the Methods section.
3. Please refer to my original question, in which I asked ‘how’, not ‘why’ were participants selected from the pool or respondents i.e., i.e., how did authors aim “to maximize variation of socio-demographic characteristics (age, sex, education level) in the sample”

Authors' response
Thank you for your comment. We included the information of S5 table in the text again (p. 9, l. 224-233).
Regarding the selection process, we describe the process more clearly now: “After voluntarily expressing their interest to participate via e-mail, potential participants received an informational letter on the procedure and conditions, and a consent form along with a short questionnaire on inclusion criteria and sociodemographic characteristics (age, gender, education level). Inclusion criteria were sufficient German language skills, legal age, interest in psychology and no degree in psychology (only laypeople) or a master’s degree in psychology and being psychological counsellors and/or psychological therapists (only psychological practitioners) or professional involvement with science communication including psychological topics (only science communicators). No other characteristics were collected that could indicate any attitudes toward PLSs. For laypeople, the number of submitted consent forms exceeded the number of available spots. While we did not employ stratified sampling or use a recruitment panel, we aimed to maximize variation in socio-demographic characteristics (i.e. a balanced gender distribution, different age decades and inclusion of the few participants with lower education levels) by purposively selecting individuals to achieve a heterogeneous sample. For psychological practitioners or science communicators, the number of submitted consent forms did not exceed the number of available spots.” (p. 9, l. 235-251)

Reviewer comment – revision 3

Thank you for response, however I note that the inclusion of the addition detail regarding the recruitment process (p9, lines 235-251) appeared in revision 2 and as such were not written in response to my repeated requests for information to clarify ‘how’, not ‘why’ were participants selected from the pool or respondents i.e., i.e., how did authors aim “to maximize variation of socio-demographic characteristics (age, sex, education level) in the sample”.

Additionally, I recommend clarifying the following:

- What is meant by “sufficient German language skills”
- What is meant by “legal age” as this may vary between countries

This paragraph is quite difficult to follow. I is long and the section relating to an interest in psychology, degree in psychology, science communication etc is not clear.

I recommend using formatting the inclusion criteria as a list instead of a paragraph.

**Item 4**

Reviewer comment – revision 1
Table 1 – Participant characteristics. I suggest the authors remember that this paper is likely to appeal to an international audience, so providing context for education levels would be helpful.

Author response
To provide context without going beyond the scope, we have classified educational levels now as low, moderate and high, and summarised the categories qualification for university and university degree in Table 1.

Reviewer comment – revision 2
I appreciate the effort the reclassify educational qualifications, however, I do not think that the using low, moderate and high is a good choice in terms of language. It could be alienating for some readers.

I also note that Table 1 appears to have moved to the Methods section. Since it reports study findings, it should be in the Results section.

Authors' response
Thank you for your comment. We partially reclassified the education levels according to the UNESCO International Standard Classification of Education. We have also rearranged Table 1 in the Results section and included a new short paragraph titled “Participants’ characteristics” (p. 13, l. 310-14).

Reviewer comment – revision 3

I note the addition of a short paragraph on p13 titled “Participant characteristics”. The purpose of this should be to summarise the key/notable findings in Table 1, and include results for age such as mean, range and median. Table 1, should only report frequency for the characteristic of ‘age’ for each age range, not median, range and mean.

**Item 5**

Reviewer comment – revision 1
Line 192 – I would have liked to review the three PLSs that were part of the interviews, however S3 file was only provided in German. I am fluent in English only.

Author response
We have now provided an English translation of the three PLS in S4 File. Please note that this is a translated version of the original PLSs provided for informational purposes only. Due to the nature of plain language summaries, the exact content and complexity (compared to scientific language) of the original cannot be fully reproduced in translation.

Reviewer comment – revision 2
Thank you for providing the PLSs translated into English as requested. I would have also liked to see the guidelines in English, but acknowledge that I did not request these.

Authors' response
We appreciate the reviewer’s interest in the guideline. We fully agree that making it available in English would be valuable. However, due to time constraints, we are unable to provide an English version at this stage. We plan to translate the guideline and make it publicly available next year.

Reviewer comment – revision 3

Noted with thanks.

**Item 6**

Reviewer comment - original
Line 707 – This statement is unsupported - “For example, it is possible that people with a lower level of education did not feel motivated to participate in the study”.

Author response - original
We reformulated this sentence so that it is clear that this is only one possible hypothetical explanation: “For example, there is a possibility that it is mainly higher educated people who are interested in taking part in psychological studies on psychological plain language summaries – and that it is also these people who are more likely to read psychological PLSs.” (p.37, l.758)

Reviewer comment – follow up 1
I appreciate that this statement has been reworded, however it still needed to be cited with supporting evidence.

Authors' response follow up 1

We added a citation of a study investigating education bias to strengthen our reasoning (p. 36, l. 734-737).

Reviewer comment - revision 3

Although there have clearly been additional references added as the total number of references has increased from N=31 in revision 2 to N=36 in revision 3, there are two issues with the use of references in this section (p36, lines 734-737).

1. There are zero references used in any of these lines to support the statements made by the authors The following sentences require references:

“However, the aim of PLSs is to help people understanding scientific research rather than supporting individual decisions with a complete presentation of options and implications. Psychological practitioners, however, considered the PLSs to be valuable informational material to be shared with interested patients.”

2. The references cited at the end of this paragraph i.e., [7, 36] were not newly added to this version, appearing in revision 2.

I see no evidence that authors have provided a reference in this section as they stated in their response.

**Item 7**

Reviewer comment – revision 2

When introducing the acronym (PLSs) for plain language summaries, all letters should be lower case, unless used at the start of a sentence i.e., plain language summaries (PLSs). This is in keeping with the established convention of other published research in the field, including the cited work by Stoll et al., 2022.

Authors' response
The term is written now in lowercase when we introduce it (p. 4, l. 80).

Reviewer comment – revision 3

Noted.
